# Modified Discrete Grey Wolf Optimizer Algorithm for Multilevel Image Thresholding

**DOI:** 10.1155/2017/3295769

**Published:** 2017-01-03

**Authors:** Linguo Li, Lijuan Sun, Jian Guo, Jin Qi, Bin Xu, Shujing Li

**Affiliations:** ^1^School of Computer, Nanjing University of Posts and Telecommunications, Nanjing 210003, China; ^2^College of Information Engineering, Fuyang Normal University, Fuyang 236041, China; ^3^School of Internet of Things, Nanjing University of Posts and Telecommunication, Nanjing 210003, China

## Abstract

The computation of image segmentation has become more complicated with the increasing number of thresholds, and the option and application of the thresholds in image thresholding fields have become an NP problem at the same time. The paper puts forward the modified discrete grey wolf optimizer algorithm (MDGWO), which improves on the optimal solution updating mechanism of the search agent by the weights. Taking Kapur's entropy as the optimized function and based on the discreteness of threshold in image segmentation, the paper firstly discretizes the grey wolf optimizer (GWO) and then proposes a new attack strategy by using the weight coefficient to replace the search formula for optimal solution used in the original algorithm. The experimental results show that MDGWO can search out the optimal thresholds efficiently and precisely, which are very close to the result examined by exhaustive searches. In comparison with the electromagnetism optimization (EMO), the differential evolution (DE), the Artifical Bee Colony (ABC), and the classical GWO, it is concluded that MDGWO has advantages over the latter four in terms of image segmentation quality and objective function values and their stability.

## 1. Introduction

Image segmentation involves the technique and process of segmenting an image into several particular unique areas and extracting useful or interested targets [1]. These areas or targets are the keys for image analysis and understanding. With the in-depth research of image segmentation technology, image segmentation techniques have been widely applied in various fields, such as medical analysis [2], image classification [3], object recognition [4], image copy detection [5], and motion estimation [6].

In recent years, many researchers have conducted massive research on image segmentation. However, there has been no theory of segmentation so far which is universally applicable. There are many algorithms for image segmentation, and classical ones are classified as algorithms based on threshold, edge, area, and others which are combined with other specific theories [7, 8]. As a commonly used image segmentation algorithm, threshold segmentation selects proper threshold to divide image into different areas or classes. Numerous different thresholding approaches have been reported in the literature. Basically, thresholding methods fall into two kinds: parametric and nonparametric [9, 10]. Parametric methods are time-consuming and computationally expensive, while nonparametric methods try to determine the optimal threshold value by optimizing some standards [10]. By introducing the optimization methods, nonparametric methods reduce the time consumption and computation and show better robustness and accuracy. Based on the above analysis, the paper will take nonparametric methods to analyze and study multilevel image segmentation.

It has proved to be feasible to determine the optimal threshold value by analyzing the histogram characteristics or optimizing objective functions [9]. These nonparametric methods can be achieved by optimizing objective functions. The commonly used optimization functions include maximization of the entropy [11], maximization of the between-class variance (e.g., Otsu's method) [12], the use of the fuzzy similarity measure [13], and minimization of the Bayesian error [14]. Among them, Kapur's optimal entropy threshold method does not require prior knowledge, which can obtain desirable segmentation result for the nonideal bimodal histogram of images which make it the most widely used method [4]. All of these techniques were originally used for bilevel thresholding and then extended to multilevel thresholding areas. However, after these methods are used for multilevel thresholding (MT), the computational complexity grows exponentially. Therefore, numerical evolutionary and swarm-based intelligent optimizations are much preferred in MT [3].

Optimization algorithm [15] is mainly used to solve the problem of the option of the threshold value and reduce the time consumption from the increase of the number of the thresholds. Genetic algorithm (GA) [16] is an early method used in the image thresholding. With the constantly emerging of the optimization algorithms, a large number of MT methods based on optimization algorithms follow. Fujun et al. [17] put forward an improved adaptive genetic algorithm (IAGA) image segmentation method; this method can adjust control parameters adaptively according to the size of individual fitness and dispersion degree of the population, which keeps the diversity of the population and improves the convergence speed; evolutionary algorithms which are inspired by swarm behavior such as Particle Swarm Optimization (PSO) [18] and artificial colony algorithm (ABC) [19] are also widely used in image segmentation problem. Oliva et al. [20] used EMO algorithm for MT problem and also applied HAS algorithm [17] to MT tasks; there are many other optimization algorithms which are also used to deal with this kind of problem and the results are also satisfactory, such as DE, CS, BF, and FFA [21–25].

As a newly proposed optimization algorithm, the GWO [26] algorithm mimics the leadership hierarchy and hunting mechanism of grey wolves in nature. Four types of grey wolves (*α*, *β*, *δ*, *ω*) are employed as the leadership hierarchy. The main steps are hunting, searching for prey, encircling, and attacking. Compared to well-known evolutionary-based algorithms such as PSO, GSA, DE, EP, and ES, the GWO algorithm shows better global convergence and higher robustness. Moreover, the GWO has high performance in solving challenging problems in unknown search spaces, and the results on semireal and real problems also prove that GWO can show high performance not only on unconstrained problems but also on constrained problems [26]. This paper, by making an analysis of GWO, tries to determine the optimal threshold for image segmentation, discretizes the continuous GWO algorithm, and then proposes modified discrete GWO algorithm. Original GWO algorithm mainly solves the problem of continuity, but the image thresholding is a discrete problem for different thresholds; therefore, GWO algorithm has to be discretized. In addition, this paper has also improved the wolves attack strategy (i.e., determining the optimal solution). While the original GWO used the average of the optimal three wolves as the best solution, the proposed algorithm in this paper abandons the average optimization strategy in the process of determining the optimal solution, and calculates the different weights on the basis of wolves fitness function and, at the same time, gives the highest weight to the dominant wolf so as to improve the convergence. The experimental results show that the algorithm determines the appropriate thresholds quickly and has better segmentation effect, high efficiency, and accuracy. Finally, the simulation experiment verifies the superiority of MOGWO. Moreover, it is the first time that MDGWO algorithm is applied to multilevel image segmentation.

The rest of the paper is organized as follows: Section 2 introduces Kapur's entropy and related work of intelligent optimization in the field of MT.  Section 3 presents the formulation of MT and Kapur's entropy objective function. The detailed process and pseudocode of the initializing, encircling, hunting, and attacking behaviors in MDGWO are presented in Section 4.  Section 5 analyzes the superiority of MDGWO based on numerous experiments in combination with Figures and Tables.  Section 6 concludes.

## 2. Related Works

In recent years, image segmentation methods based on intelligent optimization takes Otsu's method, between-class variance, Tsallis entropy, and Kapur's entropy for objective functions. These methods optimized the threshold through optimization algorithm and obtained better results on image segmentation [4]. Moreover, Akay [27] compared ABC with PSO by employing between-class variance and Kapur's entropy as objective functions. Kapur's entropy-based ABC showed better performance when the number of thresholds increases and reduced time complexity. Bhandari [28] et al. conducted comparative analysis in detail between Kapur's, Otsu, and Tsallis functions. The results show that, in remote sensing image segmentation, Kapur's entropy-based algorithm performs better than the rest generally. Ghamisi [29] et al. analyzed the performances of Particle Swarm Optimization (PSO), Darwinian Particle Swarm Optimization (DPSO), and Fractional-Order Darwinian Particle Swarm Optimization (FODPSO) in MT. By comparing them to Bacteria Foraging (BF) algorithm and genetic algorithms (GA), PODPSO shows better performance in overcoming local optimization and computational speed. Electromagnetism was introduced for MT by Horng [19], which compared it to Kapur's entropy and Otsu's method, respectively. The experimental results show that Kapur's entropy is more efficient. Before that, they verified the same test experiment through Harmony Search Optimization and obtained similar results [20]. In our previous work [30], we also take Discrete Grey Wolf Optimizer (GWO) as the tool, with fuzzy theory and fuzzy logic to achieve image segmentation. Compared with EMO and DE, our method shows better performance in segmentation quality and stability. Based on the above analysis, the algorithm which takes Kapur's entropy for objective function shows better performance. By taking Kapur's entropy as the optimization goal, the paper analyzes and studies the application of GWO in MT.

Wolf Pack Algorithm (WPA) is a new swarm intelligent method proposed by Wu et al. in 2013 [25–29, 31–33]. According to the wolf pack intelligent behavior, the researchers abstracted three intelligent behaviors, scouting, calling, and besieging, and two intelligent rules, winner-take-all generation rule of lead wolf and stronger-survive renewing rule of wolf pack. The experiments show that WPA has better convergence and robustness, especially for high-dimensional functions. In the same year, Q. Zhou and Y. Zhou [34] proposed Wolf Colony Search Algorithm based on Leader Strategy (LWCA). The idea of the algorithm originated from the phenomenon that there exists individual competitions among the wolf pack. The strongest wolf was selected as the leader of the wolves; the wolves hunted prey under the leadership of the leader, so that they could be more effective in capturing prey. The experiments show that the algorithm has better performance on convergence speed and accuracy, and it is difficult to trap-in local minimum. Coincidentally, Mirjalili et al. [26] proposed grey wolf optimizer (GWO) inspired by grey wolves in 2014. In GWO algorithm, The *α* wolf is also called the dominant wolf, the level of other three types decreases in turn, and the *ω* is the lowest-level wolf. In addition, the three main steps of hunting, searching for prey, encircling prey, and attacking prey, are implemented. Compared to well-known heuristics such as PSO, GSA, DE, EP, and ES [35–38], the GWO algorithm shows better convergence and higher local optima avoidance. In 2014, Muangkote et al. [39] proposed an improved grey wolf optimizer method (IGWO). The strategy on parameter selection of IGWO improves the search capability and the hybridization strategy increases the diversity of the agent. Zhu et al. [40] proposed to combine GWO with difference algorithm for solving the global optimization problem in 2015.

By introducing MDGWO to MT, the paper solves the problem of thresholds option by taking Kapur's entropy for objective function. The proposed algorithm shows better segmentation result, high efficiency and accuracy, and stability of the range of threshold.

## 3. Formulation of the Multilevel Image Thresholding

MT needs to set a set of threshold values *t*_*i*_; based on that, the image can be segmented into different regions. By means of intelligent optimization to obtain the optimal threshold value, the process of image segmentation has to be formulated before taking image elements or image features as parameters, to determine the optimized objective functions with the purpose of getting close to the optimal threshold value.

### 3.1. Pixel Grouping Based on Thresholding

Supposing that each image has *L* grey levels, the thresholding conversion is a process in which the pixels of image are divided into different classes or groups according to the grey levels. This kind of classification has to choose a threshold (th) or follow the following rules:

(1) C1⟵pif  0≪p<th,C2⟵pif  th≪p<L−1,

where *p* indicates the grey level of a pixel in image *I*_*g*_, *p* = {0, 1, 2,…, *L* − 1}. *C*_1_, *C*_2_ is the class of pixel *p* and th is the threshold.

Equation (1) is the description of bilevel thresholding. For MT problem, the description is

(2) $$\begin{array}{c} C_{1}⟵p\;\text{if}\;\;0\ll p<{\text{th}}_{1}\\ C_{2}⟵p\;\text{if}\;\;{\text{th}}_{1}\ll p<{\text{th}}_{2}\\ \vdots \\ C_{i}⟵p\;\text{if}\;\;{\text{th}}_{i-1}\ll p<{\text{th}}_{i}\\ \vdots \\ C_{k+1}⟵p\;\text{if}\;\;{\text{th}}_{k}\ll p<L-1, \end{array}$$

where {th_1_, th_2_,…, th_*i*_, th_*i*+1_, th_*k*_} indicates different thresholds. Therefore, MT can be described as the problem of solving the set of th. Kapur's entropy is a well-known method used to solve this kind of problem by maximizing the objective function to determine the optimal threshold.

### 3.2. Concept of Kapur's Entropy for Image Segmentation

Kapur's entropy is one of the early methods used in bilevel thresholding, and it has been applied in MT field by scholars. Kapur's entropy is an effective image segmentation technique based on threshold and probability distributions of image histogram. When the optimal threshold is allocated correctly, the entropy is the biggest of all. Entropy is used to measure the compactness and separability between classes. The purpose of the method is to find the optimal threshold and produce the maximum entropy. This method extracts the brightness level *L* from a greyscale image or a RGB image. The probability distribution of brightness value is calculated as follows:

(3) Phia=hiaSP,∑i=1SPPhia=1,a=1,2,3,if  RGB  Image,1,if  Grayscale  Image,

where *i* indicates a specific brightness level, ranging from 0 to *L* − 1, parameter *a* is used to judge whether the image is a grey image or a RGB image, SP is the total of pixels, and *h*_*i*_^*a*^ is the pixel number of the brightness level *i* in *a*. For the simplest segmentation, there are two classes defined as follows:

(4) C1=Ph1aω0ath,…,Phthaω0ath,C2=Phth+1aω1ath,…,PhLaω1ath,

where *ω*_0_(th), *ω*_1_(th) are the probability distribution of *C*_1_, *C*_2_, respectively; the equation is as follows:

(5) ω0ath=∑i=1thPhia,ω1ath=∑i=th+1LPhia.

Therefore, the objective function of Kapur's entropy can be defined as

(6) fth=H1a+H2a,a=1,2,3,if  RGB  Image,1,if  Grayscale  Image,

where entropy *H*_1_ and entropy *H*_2_ are derived by (4):

(7) H1a=∑i=1thPhiaω0aln⁡Phiaω0a,H2a=∑i=th+1LPhiaω1aln⁡Phiaω1a,

where *P*_*h*_*i*_^*a*^_ is the probability distribution of strength grades by (3) and *ω*_0_(th), *ω*_1_(th) are the probability distribution of *C*_1_, *C*_2_, respectively.

Naturally, the entropy-based method can be extended to multithresholding method. In this case, image can be divided into *k* classes with *k* − 1 thresholds. Therefore, multilevel thresholding objective function can be defined as follows:

(8) fTH=∑i=1kHia,a=1,2,3,if  RGB  Image,1,if  Grayscale  Image,

where TH = [th_1_, th_2_,…, th_*k*−1_] is a vector containing multiple thresholds and each entropy is calculated with the corresponding threshold, respectively. And (7) can be extended to the calculation of *k* entropies as follows:

(9) $$\begin{array}{c} H_{1}^{a}=\overset{{th}_{1}}{\underset{i=1}{\sum }}\frac{P_{h_{i}^{a}}}{\omega_{0}^{a}}\ln\left(\;\frac{P_{h_{i}^{a}}}{\omega_{0}^{a}}\;\right),\\ H_{2}^{a}=\overset{{th}_{2}}{\underset{i={th}_{1}+1}{\sum }}\frac{P_{h_{i}^{a}}}{\omega_{1}^{a}}\ln\left(\;\frac{P_{h_{i}^{a}}}{\omega_{1}^{a}}\;\right),\\ \vdots \\ H_{k}^{a}=\overset{L}{\underset{i={th}_{k-1}+1}{\sum }}\frac{P_{h_{i}^{a}}}{\omega_{k-1}^{a}}\ln\left(\;\frac{P_{h_{i}^{a}}}{\omega_{k-1}^{a}}\;\right), \end{array}$$

where the probabilities of *k* classes are calculated by (10); finally, it needs to categorize the pixels into corresponding classes and complete the multilevel image segmentation by (2):

(10) $$\begin{array}{c} \omega_{0}^{a}\left(\;th\;\right)=\overset{{th}_{1}}{\underset{i=1}{\sum }}P_{h_{i}^{a}},\\ \omega_{1}^{a}\left(\;th\;\right)=\overset{{th}_{2}}{\underset{i={th}_{1}+1}{\sum }}P_{h_{i}^{a}},\\ \vdots \\ \omega_{k-1}^{a}\left(\;th\;\right)=\overset{L}{\underset{i={th}_{k-1}+1}{\sum }}P_{h_{i}^{a}}. \end{array}$$

As mentioned above, multilevel thresholding is formulated to maximize Kapur's entropy, and the objective function is shown in (8). As previously mentioned, this paper will use the MDGWO to optimize the objective function; the optimization algorithm is the key to the quality of image segmentation.

## 4. Image Segmentation Based on MDGWO

### 4.1. Standard Grey Wolf Optimizer

Grey wolfs (*Canis lupus*) belongs to Canidae family, which are considered as apex predators, meaning that they are at the top of the food chain. They have a very strict social dominant hierarchy. The algorithm divides the wolves into four types: *α*, *β*, *δ*, and *ω*. The social behavior of each type wolves can be summarized as follows.

The leaders are a male and a female, called alpha. They are the most brilliant wolves and the best in terms of managing the pack. The alpha wolf is also called the dominant wolf since his/her orders should be followed by the pack unconditionally. The location of alpha presents the best solution of the problem.

The second level in the hierarchy of grey wolves is beta. The betas are subordinate wolves that help the alpha in decision-making or other pack activities. The beta wolf should respect the alpha but commands the other lower-level wolves as well. It plays the role of an advisor to the alpha and discipliner for the pack. The beta reinforces alpha's commands throughout the pack and gives feedback to the alpha.

The third level in the hierarchy of grey wolves is delta. Delta wolves have to submit to alphas and betas, but they dominate the omega, scouts, sentinels, elders, hunters, and caretakers who belong to this category. They are responsible for watching the boundaries of the territory, warning the pack in case of any danger, protecting and guaranteeing the safety of the pack, helping the alphas and betas when hunting prey, and providing food for the pack and caring for the weak, ill, and wounded wolves in the pack.

The lowest ranking grey wolf is omega. It may seem the omega is not an important individual in the pack, but it has been observed that the whole pack face internal fighting and problems in case of losing the omega, which is harmful to the group structure.

In addition to the social hierarchy of wolves, group hunting is another interesting social behavior of grey wolves. The main phases of grey wolf hunting are as follows: searching for the prey; tracking, chasing, and approaching the prey; pursuing, encircling, and harassing the prey until it stops moving; attacking toward the prey.

In order to mathematically model the social hierarchy of wolves in GWO [26], the fittest solution is considered as the alpha (*α*). Consequently, the second and third best solutions are named beta (*β*) and delta (*δ*), respectively. The rest of the candidate solutions are assumed to be omega (*ω*). In the GWO algorithm, the hunting (optimization) is guided by *α*, *β*, and *δ*. The *ω* wolves follow these three wolves.

In addition to the above four abstract models, this paper proposes MDGWO based on the standard GWO settings for MT. In the improved algorithm, the corresponding relationships between grey wolf hunting and image segmentation are shown in Table 1.

### 4.2. The Initialization of MDGWO

The size of the wolf pack is assumed as SN. SN candidate solutions (the location of the wolves is the threshold values) are generated randomly in the initialization phase. Different from the GWO, the image threshold is a set of discrete integers by rounding toward zero:

(11) $$\begin{array}{c} \vec{X_{i}}=\left\lfloor \;\mathrm{rand}\left(\;SN,1\;\right)\cdot \left(\;ub-lb\;\right)+lb\;\right\rfloor , \end{array}$$

where *ub* and *lb* are the upper limit and the lower limit of parameters (namely boundaries of parameter).

After the initialization of candidate solutions, MDGWO judges whether the initial solution $\vec{X_{i}}$ is in the range of [*ub*, *lb*]. If it is, the fitness value will be calculated, otherwise the search agent will be put back in the search space (i.e., guaranteeing the initial solution in the range of [*ub*, *lb*]) by (12), and then the fitness value will be recalculated by rounding toward zero:

(12) $$\begin{array}{c} \vec{X_{i}}=\left\lfloor \;\left(\;\vec{X_{i}}\cdot \overset{-}{\left(u+l\right)}\;\right)+ub\cdot u+lb\cdot l\;\right\rfloor , \end{array}$$

where $u=\vec{X_{i}}>ub$, $l=\vec{X_{i}}<lb$.

In the all fitness values calculated by (13) of candidate solutions, MDGWO chooses three optimal candidate solutions to assign to $\vec{X_{\alpha }}$, $\vec{X_{\beta }}$, $\vec{X_{\delta }}$, and records all the fitness values and candidate functions (namely locations of the wolves).

(13) $$\begin{array}{c} Fitness\left(\;\vec{X_{i}}\;\right)=\left\{\begin{array}{cc} \frac{1}{\left(1+f\left(\vec{X_{i}}\right)\right)}, & \left(f\left(\vec{X_{i}}\right)\geq 0\right),\\ 1+\left|f\left(\vec{X_{i}}\right)\right|, & \left(f\left(\vec{X_{i}}\right)\geq 0\right), \end{array}\right. \end{array}$$

where $\vec{X_{i}}$ is one of the candidate solutions which include a set of thresholds; then $f(\vec{X_{i}})$ is calculated by Kapur's function as shown in (8).

### 4.3. Iterative Process

After the initialization, all the search agents have to update their current locations for optimize the candidate solutions over the course of iteration. In the range of the maximum iteration (Max_iter), all the update process and optimization process will be completed.

#### 4.3.1. Encircling Prey

Grey wolves encircle prey before the hunt. In the mathematical model, the wolf pack has to update the position (namely the threshold value) constantly so that they can approach the prey. In the algorithm, all the agent position updated by (15) over the course of encirclement:

(14) D→=C→·Xp→t−X→t,

(15) X→t+1=Xp→t−A→·D→,

where *t* indicates the current iteration, $\vec{A}$ and $\vec{C}$ are coefficient vectors, $\vec{X_{p}}$ is the position vector of the prey, and $\vec{X}$ indicates the position vector of a grey wolf. The vectors $\vec{A}$ and $\vec{C}$ are calculated as follows:

(16) A→=2e→·r1→−e→,

(17) C→=2r2→,

where components of $\vec{e}$ are linearly decreased from 2 to 0 over the course of iterations and $\vec{r_{1}}$, $\vec{r_{2}}$ are random vectors in [0, 1]. The detailed selection of the two vectors can be found in [26].

#### 4.3.2. The Behavior of Hunting

The hunt is usually guided by the alpha. The beta and delta might also participate in hunting occasionally. However, in an abstract search space, we have no idea about the location of the optimum (prey). In order to mathematically simulate the hunting behavior of grey wolves, it is supposed that the alpha, beta, and delta have better knowledge about the potential location of prey. Therefore, the algorithm saves the first three best solutions obtained so far and obliges the other search agents (including the omegas) to update their positions according to the position of the best search agents. The original GWO algorithm in literature [26] calculates the updated parameter of search agents by the first three best solutions and then updates the location of search agents (namely new candidate solutions) according to their average value. As for the specific formulas and the detailed calculation, please refer to literature [26]. In order to approach the best solution more quickly, the proposed algorithm in this paper improves the current best solution in solutions updating by weighting method. The update formulations are as shown in (20) and correlation coefficients are calculated by (18), (19), where *A*_1_, *A*_2_, *A*_3_ are calculated by (16).

(18) Dα→=C1→·Xα→−X→,Dβ→=C2→·Xβ→−X→,Dδ→=C3→·Xδ→−X→,

(19) X1→=Xα→−A1→·Dα→,X2→=Xβ→−A2→·Dβ→,X3→=Xδ→−A3→·Dδ→,

(20) X→t+1=w1·X1→+w2·X2→+w3·X3→,

where *w*_1_, *w*_2_, *w*_3_ are the corresponding weights, which are calculated by

(21) w1=f1F,w2=f2F,w3=f3F,

where *f*_1_, *f*_2_, *f*_3_ calculated by (13) are the corresponding fitness values of *α*, *β*, *δ*: *F* = *f*_1_ + *f*_2_ + *f*_3_. This paper emphasizes that, different from GWO updating search agents, MDGWO uses (20) and (21) to update the location of search agents by weighting method for the first time; it is also the major contribution to the improved GWO.

#### 4.3.3. Attacking Prey

**Figure 1:**
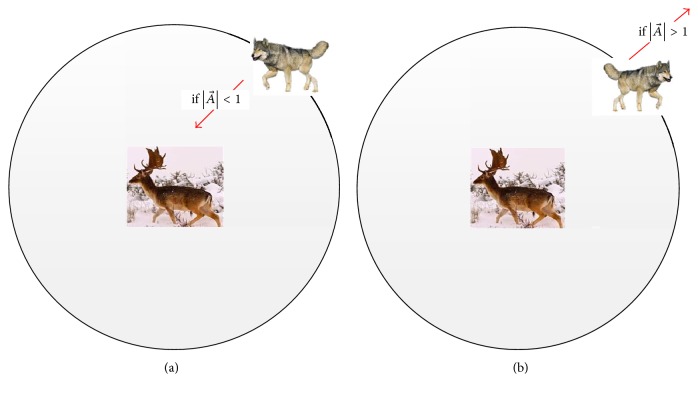
Attacking prey of grey wolf.

The grey wolves finish the hunt by attacking the prey when it stops moving. In order to mathematically model approaching the prey, we decrease the value of $\vec{e}$. Note that the fluctuation range of $\vec{A}$ is also decreased by $\vec{e}$. In other words, $\vec{A}$ is a random value in the interval [−*e*, *e*] where $\vec{e}$ is decreased from 2 to 0 over the course of iterations. When random values of $\vec{A}$ are in [−1,1], the next position of a search agent can be in any position between its current position and the position of the prey; that is, the search agent will approach the best solution gradually, as shown in Figure 1.

At the same time, for the purpose of mathematical model divergence, we utilize $\vec{A}$ with random values greater than 1 or less than −1 to oblige the search agent to diverge from the prey. As shown in Figure 1(b), $\vec{A}>1$ forces the grey wolves to diverge from the prey to hopefully find a better prey.

### 4.4. Pseudocode of MDGWO

The application of MDGWO algorithm in image segmentation mainly lies in optimizing Kapur' entropy to obtain the best threshold; therefore, the fitness function as shown in (13) will be calculated based on Kapur's entropy.

**Step 1 .**

Read image *J*; if *J* is a RGB image, then it will be processed by three channel of *J*_R_, *J*_G_, *J*_B_ and store the data in *h*^R^, *h*^G^, *h*^B^, respectively; if *J* is a grey image, then read the grey value and store it in *h*^gr^.

**Step 2 .**

According to (3), calculate image grey values and probability distribution histogram.

**Step 3 .**

Initialize the population of grey wolves, parameter $\vec{e}$, $\vec{A}$, $\vec{C}$, and Max_iter.

**Step 4 .**

Initialize the population $\vec{X_{i}}$ (*i* = 1,2,…, SN) randomly:

(22) $$\begin{array}{c} \vec{X_{i}}=\mathrm{rand}\left(\;SN,1\;\right)\cdot \left(\;ub-lb\;\right)+lb. \end{array}$$

**Step 5 .**

Use (11) to discretize $\vec{X_{i}}$ , that is, being rounded toward zero. Use (12) to adjust the singular data beyond the boundaries of search space.

**Step 6 .**

Calculate objective functions of each search agent by using (8). And calculate the fitness value of each search agent on the basis of objective functions.

**Step 7 .**

According to the fitness values, assigning first three best search agents to $\vec{X_{\alpha }}$, $\vec{X_{\beta }}$, $\vec{X_{\delta }}$, respectively.

**Step 8 .**

Updating encircling parameters based on $\vec{X_{\alpha }}$, $\vec{X_{\beta }}$, $\vec{X_{\delta }}$, which include calculating $\vec{A_{1}}$, $\vec{A_{2}}$, $\vec{A_{3}}$ by (16), $\vec{C_{1}}$, $\vec{C_{2}}$, $\vec{C_{3}}$ by (17), and $\vec{D_{\alpha }}$, $\vec{D_{\beta }}$, $\vec{D_{\delta }}$ by (18).

**Step 9 .**

Update the position of search agents based on (19) and (20) for the next hunting.

**Step 10 .**

Add one to circular point, if *q* ≥ Max_iter or meet the stop condition of the algorithm, the iteration will be finished and skip to Step 11; otherwise skip to Step 5.

**Step 11 .**

Set the location of the wolf that has the best objective function as the best threshold of segmentation.

**Step 12 .**

Input best threshold and images before and after segmentation.

## 5. Experiments and Discussion

### 5.1. Parameters Settings

**Figure 2:**
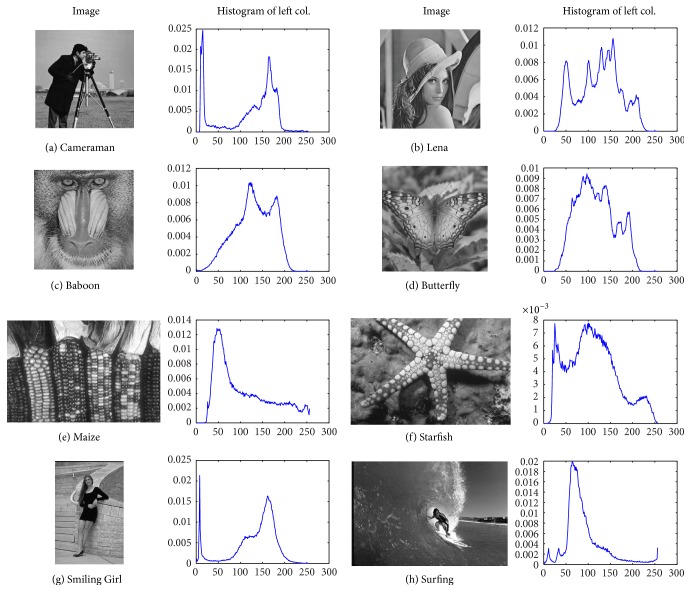
The original images and their histograms.

The proposed algorithm has been tested under a set of benchmark images. Some of these images are widely used in the multilevel image segmentation literature to test different methods (Cameraman, Lena, Baboon, and Maize). Others are chosen on purpose from the Berkeley Segmentation Data Set and Benchmarks 500 (BSD500 for short, see [41]), as shown in Figure 2. The experiments were carried out on a Lenovo Laptop with an Intel Core i5 processor and 4 GB memory. The algorithm was developed via the signal processing toolbox, image processing toolbox, and global optimization toolbox of Matlab R2011b. According to relevant literatures, many methods were proved to have certain advantages compared with previous methods. This paper chooses the best advantageous method as comparison objective; the earlier or inferior literatures will no longer be used for the analysis. Based on quantities of contrast experiments, this paper will verify the superiority of MDGWO in image segmentation by comparison of image, data, chart, and so forth. In the following sections, MDGWO will be compared with the algorithm using electromagnetism optimization (EMO) proposed in [20], the differential evolution (DE) [27], the Artifical Bee Colony (ABC) [10], and the original grey wolf optimizer. The EMO, DE, and ABC are the latest intelligent optimization methods by using Kapur's entropy so far. The comparison with GWO is mainly used to test the advantages of MDWGO.

In order to avoid the randomness of results, we use appropriate statistical metrics to compare the effectiveness of these algorithms. According to [19, 20] and the experiments, test thresholds are th = 2, 3, 4, 5 [1–3] and the stop criterion of each experiment is 150 iterations; the detailed settings of parameters are showed in Table 2.

For verifying the stability, we use (23) to calculate the standard deviation (STD) at the end of each test. Once the STD value increases, the algorithms becomes more instable correspondingly [29]:

(23) $$\begin{array}{c} STD=\sqrt{\overset{Max\_iter}{\underset{i=1}{\sum }}\frac{{\left(\theta_{i}-\varepsilon \right)}^{2}}{Max\_iter}}. \end{array}$$

In addition, the peak signal to noise ratio (PSNR [20]) is used to compare the similarity between the segmented image and original image according to the mean square error (MSE [26]) of each pixel:

(24) PSNR=20 log10⁡255MSE,  dB,

(25) MSE=∑i=1ro∑j=1coIoai,j−Ithai,jro×co,

where *I*_*o*_^*a*^ is the original image, *I*_th_^*a*^ is the segmented image, and *a* depends on the type of image (grey image or RGB image); ro, co are, respectively, the total number of rows and columns in an image.

Because Kapur's entropy is based on histogram of the image, this paper provides test images and the corresponding histograms. From Figure 2, it can be seen that each image has the only distinctive histogram which can guarantee the universality and commonality of the algorithm. More importantly, most of these histograms do not follow the bimodal characteristic; therefore, the difficulty level of optimization increases accordingly.

### 5.2. The Image Segmentation Result Based on MDGWO with Different Thresholds

**Figure 3:**
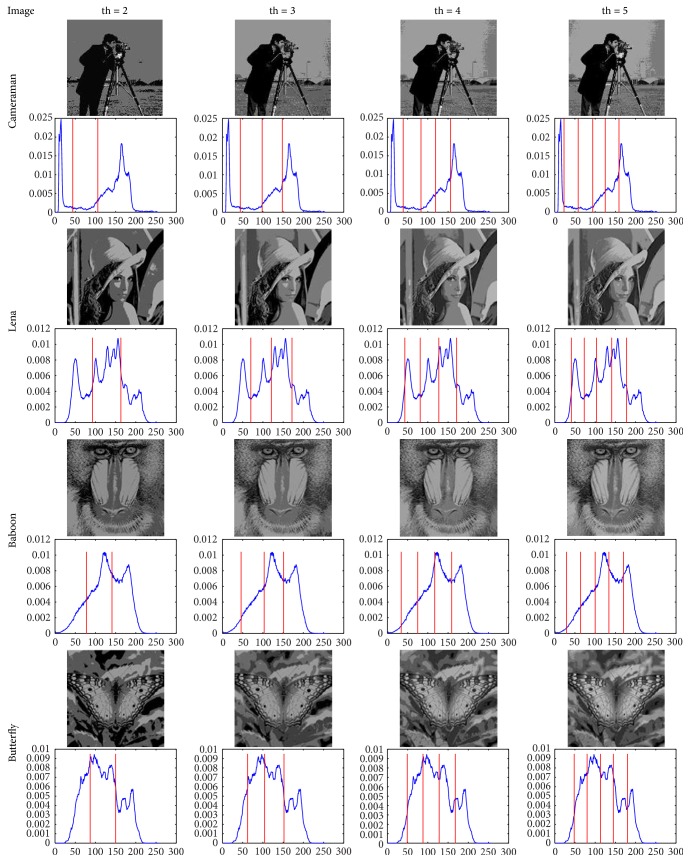
The segmentation results of (a)–(d) in Figure 2 and their thresholds in histograms.

**Figure 4:**
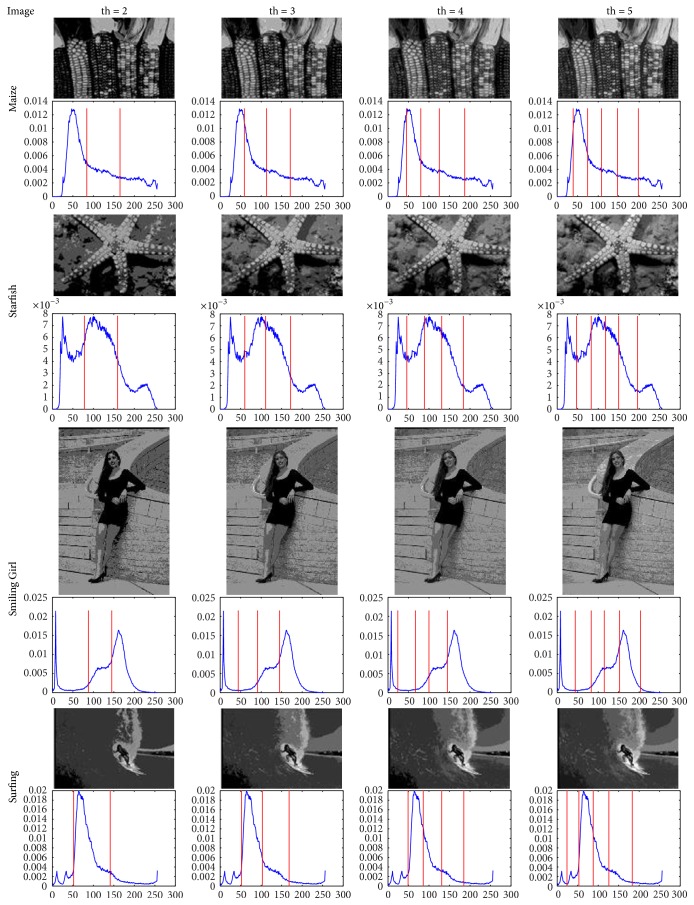
The segmentation results of (e)–(h) in Figure 2 and their thresholds in histograms.

In order to reflect the segmentation effect, Figures 3 and 4 illustrate the segmentation results of original images in Figure 2 when the threshold is 2, 3, 4, and 5, respectively. The experiments indicate that the segmentation results turn out to be finer and there are also more segmented areas when the number of thresholds is larger and vice versa. In extreme cases, when the threshold is 2, the algorithm is reduced to be binary image segmentation (foreground-background segmentation). Certainly, that how many areas are segmented is related to the applications and requirements, this method only needs to set the corresponding number of thresholds (th). Besides giving the MDGWO segmentation results, like [20], Figures 3 and 4 also mark the position of the threshold in each histogram. Compared with other MT methods, it is difficult to find the difference in terms of the segmentation effect. Therefore, Section 5.4 lists the thresholds, PSNR, STD, and MEAN for comparisons of MDGWO results and other techniques.

### 5.3. The Comparison of MDGWO in Multilevel Image Thresholding with Different Objective Functions

In Section 1, we summarize the relevant objective functions. The commonly used optimization functions include maximization of the entropy and maximization of the between-class variance (e.g., Otsu's method). In [27], the authors, respectively, analyze these objective functions and compare Kapur and Otsu systematically. The experiment results show that Kapur's entropy gets the best effectiveness. Therefore, in order to verify MDGWO's efficiency, we compare the thresholds and PSNR between Otsu and Kapur in Table 3.

As shown in Table 3, the threshold distribution of Kapur is more dispersed and wider in most cases. However, the effect of image thresholding cannot be seen from the threshold distribution directly. Therefore, we focus on analysis the PSNR between Otsu and Kapur. In terms of PSNR, the Kapur method produces higher PSNR values on most items in Figure 2, except for the Sea Star Image with *k* = 3,5 and Surfer Image with *k* = 4.

The average value of Kapur is improved averagely by 22.24% compared to Otsu by checking against the corresponding PSNR values which are obtained from the MDGWO. The maximum value is increased by 53.42% for Cameraman Image when *k* = 3. Therefore, the Kapur method is significantly better than the Otsu method, and we also mainly analyze the effectiveness of Kapur's method in Section 5.4.

### 5.4. The Comparison of Quality Assessment by PSNR, STD, and MEAN

This paper adopts PSNR standard to evaluate the segmentation quality in comparison with other MT methods. Tables 4–6 illustrate the PSNR values under different thresholds of MDGWO, GWO, EMO, DE, ABC, and the MEAN and STD of objective functions. As shown in Table 4, it can be seen from the PSNR values that MDGWO gets the highest evaluation in most cases. Thus, it proves that MDGWO gets better results on image segmentation. In addition, when the number of thresholds increases, it shows superior PSNR value. In detail, the MDGWO algorithm produces higher PSNR values on all items in Table 4 except for the Smiling Girl Image with *k* = 5 and Surfer Image with *k* = 4 on the result of DE.

As shown in Table 4, the average value of MDGWO is improved averagely by 13.16% compared to GWO by checking against the corresponding PSNR values. The maximum value is increased by 45.05% for Butterfly Image when *k* = 5.

Comparing with MTEMO, the average value of MDGWO is improved averagely by 11.56% by checking against the corresponding PSNR values. The maximum value is increased by 39.94% for Surfer Image when *k* = 2.

By comparing with DE, the average value of MDGWO is improved averagely by 38.14% by checking against the corresponding PSNR values. The maximum value is increased by 97.89% for Baboon Image when *k* = 2. The PSNR of the Smiling Girl with *k* = 5 and Surfer Image with *k* = 4 are slightly lower, but the gap is not significant.

Comparing between ABC and MDGWO, the average value of MDGWO is improved averagely by 10.55% by checking against the corresponding PSNR values. The maximum value is increased by 38.36% for Surfer Image when *k* = 2.

From the perspective of STD which is shown in Table 5, it can be observed that MDGWO shows obviously advantages over MTEMO, DE, ABC, and GWO. The smaller the STD is, the smaller the change of fitness functions over the course of iterations will be, that is, the better stability of segmentation. Therefore, the results show that MDGWO obtains exciting effect in stability. So MDGWO's stability is better.

Compared with GWO, the average value of MDGWO is improved averagely by 73.52% compared to GWO by checking against the corresponding STD values. The maximum value is increased by 96.44% for Baboon Image when *k* = 3, and the minimum value is increased by 24.92% for Baboon Image when *k* = 3.

By comparing with MTEMO, the average value of MDGWO is improved averagely by 47.88%. The maximum value is increased by 87% for Lena Image when *k* = 2, and the minimum value is increased by 0.6% for Surfer Image when *k* = 3.

Comparing between DE and MDGWO, the average value of MDGWO is improved averagely by 95.60%. The maximum value is increased by 99.21% for Baboon Image when *k* = 2. The minimum value is increased by 88.32% for Butterfly Image when *k* = 3.

Comparing with ABC, the average value of MDGWO is improved averagely by 45.90%. The maximum value is increased by 79.70% for Lena Image when *k* = 3, and the minimum value is increased by 12.93% for Baboon Image when *k* = 5.

As far as MEAN is concerned, this parameter presents the average value of fitness function over the course of iterations in Table 6, which reflects the algorithm stability to some extent. But its accuracy of evaluation is relatively low which can only reflect the fuzzy stability of the algorithm. The experiment data is offered here just in response to the parameter provided in literature [20]. In comparison with GWO, MTEWO, DE, and ABC in Table 6, it can be safely assumed that, in most cases, MDGWO obtains higher MEAN of fitness function. Moreover, the difference is extremely small when MDGWO's MEAN is lower.

From the analyses of Tables 3–6, together with the visual effects of Figures 2, 3, and 4, it can be observed that MDGWO method obtains better segmentation effect and has advantage in optimal process, accuracy, stability, and robustness. Therefore, MDGWO is a MT algorithm with high accuracy and high segmentation quality.

## 6. Conclusion

This paper proposes a modified discrete grey wolf optimizer, which is used to optimize the image histograms and realize the multilevel image segmentation. Based on the high efficiency of GWO in the course of optimization and stability, this paper successfully applies the MDGWO to the field of MT by improving the location selection mechanism of *α*, *β*, and *δ* during the hunting and using weight to optimize the final position of prey (best threshold). The MDGWO method not only obtains better segmentation quality but also shows obvious superiority over GWO, MTEMO, DE, and ABC in stability, accuracy, and multilevel thresholding.

## Figures and Tables

**Table 1:** The corresponding relationships between MDGWO and image segmentation.

MDGWO	Image segmentation
Positions	Threshold segmentation solution
Alpha_Pos	Optimal solution
Alpha_score	The largest fitness value
Fitness	Fitness function value
Best_R	Best fitness

**Table 2:** Parameters settings of MDGWO.

Parameters	Population size	Threshold	Number of iterations	Run time	Lower bound	Upper bound
Value	50	2, 3, 4, 5	150	35	1	256

**Table 3:** The comparison of MDGWO's efficiency between Otsu and Kapur.

Image	*k*	Otsu thresholds					PSNR	Kapur thresholds					PSNR
Cameraman	2	70	144				11.9475	44	106				14.4629
	3	58	119	156			12.9933	44	98	148			19.9337
	4	40	93	139	170		16.0180	39	83	119	156		21.1684
	5	35	81	121	149	174	16.3710	23	58	94	125	159	23.1191

Lena	2	92	151				13.1029	93	163				14.7164
	3	80	126	171			15.7626	70	121	172			17.5144
	4	75	114	145	180		16.4291	43	81	127	171		20.1729
	5	73	109	136	161	189	16.7206	41	73	103	141	178	21.8208

Baboon	2	97	149				12.4396	78	141				16.0302
	3	83	123	160			13.7731	46	103	151			18.6340
	4	70	105	136	167		14.9493	34	75	117	159		20.5198
	5	66	98	125	150	175	16.0012	29	64	100	134	170	22.0929

Butterfly	2	100	152				12.4400	87	150				14.9325
	3	81	118	159			14.2563	62	104	152			18.5539
	4	73	101	129	164		14.6987	49	88	128	168		21.0711
	5	69	95	121	149	177	16.4688	48	80	113	145	180	22.4539

Maize	2	92	168				13.6677	84	165				14.0407
	3	76	128	187			15.2676	59	113	171			16.5853
	4	65	104	150	200		16.7309	45	80	125	187		18.9818
	5	56	86	123	164	208	18.5649	39	74	108	147	198	20.8792

Sea Star	2	85	157				14.8445	81	160				14.8595
	3	69	120	178			17.6040	60	111	172			17.4583
	4	59	99	137	187		19.4039	46	89	131	184		19.4487
	5	51	85	117	150	193	21.4000	47	83	118	150	196	20.9681

Smiling Girl	2	60	123				13.1358	88	145				18.0033
	3	47	100	131			12.8470	44	91	145			19.1148
	4	28	74	111	136		15.6914	24	67	100	145		20.0759
	5	20	63	95	124	158	18. 2260	44	83	115	152	203	21.7937

Surfer	2	92	162				12.5401	52	141				16.4343
	3	71	111	177			16.0569	52	103	168			18.9343
	4	47	81	118	179		20.8786	49	86	131	185		20.7218
	5	46	77	105	143	196	21.6071	24	53	88	126	183	21.8961

**Table 4:** PSNR metrics of MTEMO, DE, ABC, GWO, and MDGWO.

Image	*k*	MTEMO	DE	ABC	GWO	MDGWO
Cameraman	2	13.626	12.584	13.920	14.279	14.463
	3	18.803	17.584	14.462	19.696	19.934
	4	20.586	20.111	20.81	20.809	21.168
	5	20.661	21.282	22.40	22.404	23.119

Lena	2	14.672	8.823	14.590	14.680	14.716
	3	17.247	14.386	17.197	17.416	17.514
	4	18.251	16.151	18.559	19.762	20.173
	5	20.019	16.720	20.321	21.299	21.820

Baboon	2	16.016	8.103	16.007	16.024	16.035
	3	16.016	12.596	18.592	18.632	18.634
	4	18.485	13.178	18.417	20.480	20.519
	5	20.507	13.135	20.224	22.060	22.092

Butterfly	2	11.065	8.702	14.402	14.762	14.932
	3	14.176	13.028	14.504	17.873	18.553
	4	16.725	13.028	16.189	21.021	21.071
	5	19.026	14.786	19.286	21.485	22.453

Maize	2	13.633	10.549	13.590	13.950	14.040
	3	15.229	13.022	15.295	16.201	16.585
	4	16.280	14.270	16.346	18.713	18.981
	5	17.211	15.079	17.046	20.410	20.879

Sea Star	2	14.398	8.610	14.395	14.809	14.885
	3	16.987	14.078	16.981	17.431	17.458
	4	18.304	16.191	18.427	19.421	19.448
	5	20.165	16.474	20.330	20.887	20.968

Smiling Girl	2	13.420	14.986	13.514	17.989	18.003
	3	18.254	11.243	18.069	18.742	19.114
	4	18.860	14.556	18.826	19.823	20.075
	5	19.840	22.980	19.769	21.214	21.793

Surfer	2	11.744	9.737	11.878	16.154	16.434
	3	18.584	11.638	18.762	18.895	18.934
	4	19.478	21.866	19.647	20.234	20.721
	5	20.468	19.576	20.479	21.699	21.896

**Table 5:** STD metrics of MTEMO, DE, ABC, GWO, and MDGWO.

Image	*k*	MTEMO	DE	ABC	GWO	MDGWO
Camerman	2	0.1849	1.2592	0.1235	0.1697	0.0462
	3	0.1649	1.7601	0.2122	0.2287	0.0758
	4	0.2943	2.1995	0.3003	0.3627	0.0659
	5	0.2999	2.6579	0.2784	0.4278	0.1964

Lena	2	0.0969	1.2902	0.0349	0.1536	0.0126
	3	0.1665	1.7822	0.1300	0.3570	0.0264
	4	0.2800	2.2104	0.1872	0.5965	0.0939
	5	0.2515	2.5992	0.1827	0.5946	0.1570

Baboon	2	0.0108	1.2862	0.0358	0.2171	0.0102
	3	0.0393	1.7678	0.0202	0.4376	0.0156
	4	0.1727	2.2126	0.1610	0.2986	0.1184
	5	0.2868	2.6239	0.2660	0.5377	0.2316

Butterfly	2	0.0903	1.2708	0.0750	0.2179	0.0465
	3	0.2207	1.7429	0.2952	0.2712	0.2036
	4	0.2482	2.2368	0.3906	0.4808	0.2415
	5	0.2900	2.6571	0.4818	0.5096	0.2684

Maize	2	0.0356	1.3501	0.0218	0.3571	0.0188
	3	0.1222	1.8612	0.0901	0.2225	0.0270
	4	0.2305	2.3230	0.2605	0.3903	0.0927
	5	0.2502	2.7461	0.3834	0.4584	0.1677

Sea Star	2	0.1073	1.3547	0.1088	0.1728	0.0290
	3	0.1497	1.8741	0.1752	0.2028	0.0374
	4	0.1596	2.3307	0.1817	0.5032	0.1119
	5	0.2639	2.7550	0.2180	0.4550	0.1437

Smiling Girl	2	0.0377	1.2516	0.0318	0.1834	0.0111
	3	0.0955	1.7311	0.0577	0.1712	0.0242
	4	0.1966	2.1878	0.1094	0.2508	0.0781
	5	0.1550	2.5989	0.2768	0.5273	0.1302

Surfer	2	0.0579	1.3213	0.0303	0.2681	0.0245
	3	0.1002	1.8337	0.1646	0.3014	0.0996
	4	0.3382	2.3317	0.1686	0.3162	0.1424
	5	0.3690	2.7846	0.2580	0.3815	0.1706

**Table 6:** MEAN metrics of MTEMO, DE, ABC, GWO, and MDGWO.

Image	*k*	MTEMO	DE	ABC	GWO	MDGWO
Camerman	2	17.584	12.4212	17.7638	14.634	17.471
	3	21.976	17.3764	22.3059	21.107	21.919
	4	26.586	21.7520	26.8409	24.927	27.480
	5	30.506	26.2505	30.8328	30.436	30.548

Lena	2	17.831	12.7730	17.8139	17.809	18.396
	3	22.120	17.6428	22.0832	22.074	22.856
	4	25.999	21.8784	26.0615	25.318	26.447
	5	29.787	25.7311	29.9664	29.252	30.381

Baboon	2	17.625	12.7333	17.6760	17.679	18.619
	3	22.269	17.5005	22.1276	22.129	22.949
	4	26.688	21.9010	26.3912	26.194	26.900
	5	30.800	25.9681	30.3464	30.067	30.076

Butterfly	2	16.681	12.5796	17.4205	17.425	17.723
	3	21.242	17.2545	22.2209	21.585	22.498
	4	25.179	22.0176	26.3794	25.267	26.190
	5	28.611	26.1795	30.6533	29.492	29.899

Maize	2	18.631	13.3566	18.6316	18.604	19.542
	3	23.565	18.4245	23.2496	22.941	23.939
	4	27.529	22.9813	27.3931	26.936	27.842
	5	31.535	27.1709	31.2127	31.023	30.694

Sea Star	2	18.754	13.4104	18.7295	18.321	19.587
	3	23.323	18.5516	23.2738	23.245	23.901
	4	27.582	23.0719	27.5057	27.136	28.210
	5	31.562	27.2551	31.4919	31.167	31.958

Smiling Girl	2	17.334	12.3892	17.3129	17.136	18.035
	3	21.904	17.1339	21.8601	21.253	21.980
	4	26.040	21.6541	25.9904	25.050	26.597
	5	30.089	25.7130	30.0225	29.870	30.574

Surfer	2	18.339	13.0786	18.3393	18.283	18.869
	3	23.231	18.1492	23.2889	23.243	24.135
	4	27.863	23.0548	27.8017	27.275	27.447
	5	31.823	27.4979	31.7335	31.384	31.325
